# Capital of Life *in* Death: How Bereaved Individuals Mobilise Cultural and Social Capital in UK Death Administration

**DOI:** 10.1111/1468-4446.70024

**Published:** 2025-08-06

**Authors:** Laura Towers, Kate Reed

**Affiliations:** ^1^ University of Manchester Manchester UK

**Keywords:** bereavement, cultural capital, death administration, family, relationality, social capital

## Abstract

This paper uses Bourdieu's concepts of cultural and social capital to critically examine death administration in the UK. Death administration relates to a set of tasks that bereaved individuals (usually a family member) must complete when someone dies‐such as probate, asset management and funeral planning. It is a hidden form of administration which is complex, contradictory and often challenging to complete. Drawing on data from qualitative research, the paper shows how death administration is a relational activity which requires people to draw upon and transmit different forms of cultural capital (embodied, objectified and institutional) across life and death. Such capital is strongly mediated by family, and by a bereaved individual's ability to mobilise a wider set of social networks and resources. The article concludes by highlighting the ways in which the overall volume of capital bereaved individuals possess affects their ability to successfully navigate death administration. By bringing Bourdieu's theory of cultural and social capital together in a new empirical area, and by illuminating capital transmission across the boundaries of life and death, the paper offers an original conceptual contribution. By analysing new empirical data on death administration, the paper also extends the substantive focus of research on death and dying.

## 1 Introduction

According to Bourdieu, the social position of individuals, groups, or institutions is determined by the *volume* and *composition* of capital they hold (Wacquant 2008, 268). This capital exists in different forms—economic, cultural, and social—which, when legitimated, are converted into symbolic capital and power (Benzer and Reed 2019). As the distribution of cultural and economic capital is the main source of class differentiation in society (Bourdieu 1984), research has tended to concentrate on the relationship between cultural and economic capital (Lareau and Weininger 2003; Sullivan 2002). This means there has been significantly less academic focus on the connections between cultural and social capital. Moreover, while the concept of cultural capital has been used to examine a range of areas in social life, less is known about its applicability in the context of death.

A person's death in the UK necessitates a process of administrative duties for someone connected to the deceased, usually a family member. The various tasks that must be completed include notifying officials, dispersing belongings, and managing probate. According to the UK Commission on Bereavement (2022), bereaved individuals often struggle to deal with these time‐consuming and time‐sensitive administrative responsibilities during a time of potential vulnerability and upheaval (Reed and Balazs 2024), yet death administration remains under researched (Nikkola et al. 2013; Gijzen et al. 2016). Scholars have begun to examine how limited economic capital creates challenges for bereaved individuals trying to arrange a funeral (DiGiacomo et al. 2015; Woodthorpe and Rumble 2016), highlighting the lack of practical support offered (Holloway et al. 2013; Aoun et al. 2019). Less is known, however, about the vital role that cultural and social forms of capital can play in this context. For example, what sources of knowledge do people draw on to navigate these complex administrative systems? What roles do family and social networks play?

Central to this paper is the understanding that cultural capital is cultivated, transmitted and enhanced in the context of family practices and intimacies (Silva 2005). Existing research demonstrates that funeral planning, as one aspect of death administration, is a highly relational activity (Woodthorpe and Rumble 2016) and yet (reflective of wider death studies ideology—see Mallon and Towers 2024), research on death administration is largely individualistic, failing to account for how bereaved individuals are embedded in a range of social networks. This paper adopts a novel approach by employing a relational framework to explore how bereaved individuals draw on cultural and social capital (both familial and professional) to complete a range of death administration tasks. In doing so, the article brings Bourdieu's theory of cultural and social capital together in a new empirical area and offers an original contribution to sociological research on death and dying, as well as the sociologies of families and relationships.

We begin by outlining background literature along with the project's conceptual focus and method. The main part of the paper uses qualitative data to consider experiences of death administration in the United Kingdom, with discussion of the study findings presented in three sections: cultural capital in the aftermath; relationality and the transmission of capital across life and death; and the role of institutional capital. Our analysis shows that people draw on various forms of cultural and social capital to navigate death administration, noting how a lack of capital in this context can mean that individuals miss out on accessing crucial financial resources (such as bereavement support payments). In this context, cultural capital is highly relational and strongly mediated by family, influencing a bereaved individual's ability to mobilise wider social capital and institutional resources. It is also often acquired and transmitted *tacitly* across the boundaries of life and death. The article concludes by showing how overall volume of capital (cultural, economic and social) affects the ability of bereaved individuals to successfully navigate death administration. By utilising Bourdieu's theory of capital in a novel empirical area and employing the concepts of cultural and social capital within a relational framework to problematise the boundaries between life and death, the paper provides a novel conceptual contribution.

## 2 Exploring Death Administration Through a Relational Lens

Proponents of relational sociology emphasise the ‘importance of always putting the individual in the context of their past, their webs of relationships, their possessions and their sense of location’ (Smart 2007, 45). For example, Mason (2004) emphasises the centrality of relationality in her research on residential histories, demonstrating that relationality consistently featured within her participant narratives, even for those who perceived themselves as individualistic. Roseneil and Ketokivi (2016) suggest that retaining consideration of agency is essential for discussion of relationality, as it is this which allows the individual to reflexively engage with social phenomena and critically evaluate these interactions. A relational understanding does not deny agency, rather, agency is expressed ‘always within webs of connectedness’ (Smart 2007, 187).

Though there is growing awareness of the relationality of pre and post death experiences (see Almack 2022; Borgstrom et al. 2019; Ribbens et al. 2023; Towers 2022, 2024), this relational understanding is largely absent from the current literature on death administration. Existing research on death administration tends to adopt an individualistic approach, thereby denying the volume and quality of human resource required to complete administrative duties. Yet as the authors have previously reported (Towers et al. 2023; Reed and Balazs 2024), death administration is often distributed among family members as a way of sharing the emotional and organisational load. This connects with the findings of Finch and Mason's (2000) research on inheritance, which emphasises the role of kinship and relationships in people's decision‐making around the bequeathing and collecting of inheritance.

Acknowledging the centrality of social relationships is essential, however, because there are often limitations regarding who is allowed to enact different aspects of death administration.^1^ It is usually seen as the duty of a family member, with hierarchical assumptions about who will carry out specific tasks (Bailey 2012). For example, Corden et al. (2008) note that organising a funeral is considered the responsibility of a surviving spouse following the death of a partner. While it is suggested that such hierarchies are necessary to facilitate organisational policy procedures (Robson and Walter 2012), a consequence of these structures is that responsibility can fall to those who are less well‐equipped to handle such pressures. Moreover, some deaths are more difficult to administrate, such as those subject to inquest (Towers et al. 2023), which can further burden those already struggling with death administration processes.

The relational nature of certain aspects of death administration is illustrated by Woodthorpe and Rumble (2016) in their research on funeral expenses payments. Personal assessment of who is the most appropriate person to organise and pay for a funeral does not always correspond with institutional assessment. For example, Woodthorpe and Rumble outline the case of an adult sibling who was denied a funeral payment claim for their mother's funeral, because it was assumed they could share the cost with their long‐estranged brother. This is relevant for two reasons. First, it highlights that the participant was assumed by the state to have more capital (economic, in this instance) than they did, based on assumptions of familial connection and obligation. Second, it reflects Gilding's (2010) assertion that individual reflexive choice can be bound up in institutional rules and norms, with normative political assumptions about familial obligation impacting upon individual freedoms.

Relational circumstance, therefore, has a significant influence on the level and type of capital that individuals have access to when carrying out death administration. As such, the discussion in this paper adopts a relational framework to locate individuals within their webs of connectedness. By including those who have died within these relational webs, we demonstrate how people draw upon and transmit different forms of cultural capital (embodied, objectified and institutional) across the boundaries of life and death, thereby making a novel conceptual contribution to discussion of Bourdieu's work. Moreover, by drawing upon Bourdieu's work as a theoretical lens, this paper considers the role of social and cultural capital in the completion of death administration, exploring the role of family in mediating and transferring this capital. This article also makes a substantive empirical contribution to the sociologies of death, dying and bereavement by illuminating death administration as a relational process, whilst expanding the remit of family sociology by acknowledging the role of families and relationships in pre and post death experiences.

## 3 Theorising Capital Across Life and Death

Developed by Bourdieu originally through his own empirical research in education (Bourdieu 1986; Bourdieu and Passeron 1990), cultural capital is the theoretical hypothesis that made it possible to explain the unequal academic achievements of children from different social classes. For Bourdieu, cultural capital can exist in three different forms: in the *embodied* state (dispositions of the mind and body), in the *objectified* state, in the form of cultural goods (pictures, books, dictionaries, instruments, machines etc.) and in the *institutionalised* state (Bourdieu 1986, 47). This paper draws on these aspects of Bourdieu's theory of cultural capital to examine death administration: the embodiment of cultural capital and how it is transferred tacitly across life and death, and the underpinning role that family and wider social relationships play in this process. Bourdieu's analysis of cultural capital was based on the traditional nuclear family with the father as the originator. As Silva (2005) argues, however, cultural capital exists in the context of family practices and intimacies which are not traditional because partnerships and parenting relationships in contemporary society are varied. We will adopt this wider understanding of family and the acquisition and transmission of capital in our analysis of death administration.

Bourdieu argues that embodied cultural capital cannot be accumulated past the appropriating capacities of an individual agent. It decays with the biological capacity (and memory etc.) of its owner. Furthermore, the ability to accumulate all forms of cultural capital (embodied, objectified, and institutionalised) arises from the outset only with the offspring of families endowed with strong cultural capital. Bourdieu argues that this hidden (domestic) transmission of cultural capital plays a significant role in the reproduction of different forms of capital (and subsequently an agent's position within social space) (Benzer and Reed 2019).

The concept of capital ‐ alongside habitus and field ‐ forms the organising principles of Bourdieu's work (Wacquant 1989). Habitus being the ‘durably installed generative principle of regulated improvisations” (Bourdieu 1977, 78) reflects capital, especially cultural capital. It acts as a system of durable and transposable *dispositions* through which individuals perceive, judge, and act in the world (Benzer and Reed 2019). The concept of *field* refers to a social arena (e.g., art, science, religion, economy, law and politics) within which struggles or manoeuvres take place over specific resources or stakes and access to them (Wacquant 1989). Although this paper focuses specifically on capital, we acknowledge how *dispositions* and position in a specific *field‐*as determined by capital‐can either facilitate or hinder bereaved individuals’ ability to navigate death administration in different social spaces.

Although important, cultural capital does not operate in isolation. As Edgerton and Roberts (2014) argue, Bourdieu's forms of capital (economic, cultural and social) are mutually constitutive. For example, the broader one's social networks become, the greater the opportunity for enhancing one's economic assets. In this paper, we seek to illuminate some of the intersections between cultural and social capital. Social capital refers to group association or membership which provides its individual members with the support of collectively owned capital (Bourdieu 1986). These relationships may exist only in the practical state, in material and/or symbolic exchanges which help to maintain them (Bourdieu 1986).

Our article contends that an individual's ability to navigate death administration is strongly mediated by their access to different forms of cultural and social capital acquired across life and death. In doing so it extends the use of the concept of cultural capital in several respects: firstly, because it applies the concept to a novel social phenomenon‐death administration‐ and in doing so stretches the versatility of the concept. Secondly, rather than applying the concept to substantive issues concerned with life (such as education) we unpack the ways in which cultural capital is used in death. Through this process we illuminate some of the latent ways in which capital is transmitted across and beyond traditional boundaries of life and death. Thirdly, throughout the paper we recognise the underpinning role that economic capital plays in assisting bereaved individuals to complete death administration. We aim, however, to centre our analysis on the intersection of cultural and social capital. In doing so the article offers a fresh angle to existing manifestations of the concept. Finally, by demonstrating the value of using the concepts of cultural and social capital across life and death, we will illuminate its relevance for studying wider social phenomena beyond its current sociological application.

## 4 The Study

The findings presented in this paper are taken from a 6‐month exploratory project, conducted in 2022, that sought to scope people's understanding and experiences of undertaking tasks related to death administration in the UK. Using an interview schedule developed in consultation with the National Bereavement Service,^2^ the research team carried out online video call interviews (lasting approx. 1 h each) with 20 individuals in the UK, bereaved between 2017–2022. People voluntarily responded to a call for participants advertised through the University of Sheffield and the National Bereavement Service. Participants (see Table 1 for an overview) helped to varying degrees with completing the administrative tasks that arose following the death of a relative. All were over 18 years of age, bereaved by any means within the 5 years prior to interview.

**TABLE 1:** Participant information.

Name (pseudonym)	Gender	Age	Relationship to the deceased	Relation of deceased and location of death	Year(s) of death
Clara	Female	54	Daughter	Father, hospital	2021
Ella	Female	33	Granddaughter	Grandmother, home	2017
Margaret	Female	43	Daughter	Mother, hospital	2017
Lauren	Female	47	Daughter	Mother, hospice father, home	2019/2021
Janet	Female	66	Wife	Husband, care home	2020
Teresa	Female	47	Daughter	Father, hospice	2017
Howard	Male	59	Son	Mother, care home	2019
Barbara	Female	48	Daughter	Mother, home	2022
Miriam	Female	23	Daughter	Father, hospice	2020
Veronica	Female	59	Daughter	Mother, hospital	2019
Mark	Male	64	Son	Father, home	2022
Nora	Female	54	Daughter	Mother, home	2022
Karen	Female	59	Wife	Husband, home	2021
Grace	Female	52	Daughter	Father, hospital	2019
Lily	Female	43	Daughter, sister	Father, home	2020/2021
				Sister, hospital	
Alison	Female	42	Daughter	Mother, hospital	2018
Diana	Female	55	Wife	Husband, hospice	2022
Fiona	Female	58	Daughter	Father, home	2021
Ian	Male	73	Husband	Wife, home	2021
Rose	Female	36	Daughter	Father, hospital	2019

Participants included 17 women and 3 men, living in a variety of locations across the UK. Two individuals had completed death administration tasks for two relatives within the 5‐year research time frame; these deaths are also accounted for within the project data. Participant ages ranged from 20 to 73 years, though the majority (16) were aged 42–66. Despite attempting to recruit participants from different ethnic and socio‐economic backgrounds our participants were mainly middle class (14 in senior professional roles, six in administrative roles) and majority ethnically White (19 White, 1 Asian).

While some groups (such as women) were well represented in the study, others (such as men and individuals from minoritised ethnic groups) were not. This relates to the short timeframe of the project and our inability to apply additional recruitment measures to target groups less well represented in research. This is, however, an exploratory study that does not seek to offer a generalisable account of experiences of death administration. Rather it aims to offer insight into some of the challenges individuals face when attempting to navigate death administration processes and inform future research in this area.

Interviews were designed in three parts (introduction, practical tasks/duties, and emotional experiences), though the semi‐structured approach taken meant that questions were asked in varying orders based upon participant responses. Example questions include, what were the main practical difficulties you encountered following the death of your relative? What did you find helpful during this time? To what extent did friends or family members participate in making practical arrangements? To support recall during interviews, participants were shown a map of bereavement trajectories developed by the research team. This map displayed the main steps associated with the death administration process in a one‐page document and acted as a visual prompt to aid participants when constructing their bereavement narrative. It also helped to support participants through the interview, thereby lessening the burden of trying to recall events from an emotionally turbulent time. All aspects of the conversation were recorded and transcribed, applying anonymisation techniques to maintain the confidentiality of participants.

Transcripts were analysed inductively, meaning that coding and theme development were directed by the content of the data. Applying a reflexive approach to thematic analysis (Braun and Clarke 2019), we familiarised ourselves with the data through multiple readings and coded the data using NVivo software. We then generated initial themes to be reviewed, before defining and naming those themes and writing up. Analysis focused on the three main issues identified during the initial literature review, which were: the challenges of administration at different steps of the bereavement trajectory (registering the death, organising the funeral, dividing the estate etc.); the emotional aspects of the administrative process; and the main areas for improvement as identified by those interviewed.

The study was carried out in collaboration with the National Bereavement Service, funded by UKRI Research England, and received ethical approval from the University of Sheffield. As bereaved individuals ‘rank highly among the categories of research‐vulnerable people’ Hockey (2014, 98), several steps were taken to ensure participant welfare, including: reassuring participants that they did not have to answer any questions they found distressing, offering breaks as needed, reminding them of their right to withdraw should the experience prove emotionally stressful, and signposting participants to the National Bereavement Service's support pages should they seek further assistance. The project team are experienced in conducting sensitive and emotionally demanding research and recognise the role of researcher positionality in this context (Reed and Towers 2021). Members of the team also had personal experience of bereavement, which they could draw on to conduct interviews with sensitivity and ‘empathic distance’ (Rowling 1999), and reflect on how these experiences framed the research lens (Pearce 2010).

## 5 Findings

Discussion of the study findings will now be presented across the following three sections: cultural capital in the aftermath; relationality and the transmission of capital across life and death; and the role of institutional capital. Quotations provided in this section prioritise the perspectives and understandings of the participants interviewed. There may be factual errors in people's recollections of the actions required and steps taken following a person's death, but these have been purposefully retained to highlight areas of potential misunderstanding and miscommunication during the death administration process.

### 5.1 Cultural Capital in the Aftermath

Most participants found the death administration process complex and time‐consuming. The stress of completing administrative tasks was felt in combination with an awareness of needing to be extremely organised at a time when participants felt vulnerable and disoriented (Reed and Balazs 2024). Limited organisational guidance meant responsibility for navigating these processes was deferred to individuals. As we will show in this section, cultural capital, or lack of it, can play an important role in assisting bereaved individuals in navigating death administration.

Cultural capital, as articulated by Bourdieu in his work on education, is transferred through three modes of ‘pedagogic action’. Informally, cultural capital is transmitted through *diffuse education* (interaction with others), while family education is the main source of transference of embodied cultural capital, with school providing the formal means (Bourdieu and Passeron 1990; Claussen and Osborne 2012). When it came to navigating death administration, participants often highlighted a lack of informal or formal modes of pedagogic action (i.e., they had no one to teach them what to do), as Alison explains:

There seemed to be quite a lot of onus on us to figure out what we needed to do, and remember to do things… nobody teaches you how to do this, you only know because you experience it, and then you’re in the most horrible situation and you’ve got to deal with it.

(Alison, 42, bereaved daughter)

Death administration must be figured out ‘in the moment’, under time pressure, at the point that someone is potentially feeling overwhelmed and highly vulnerable (Reed and Balazs 2024; Nikkola et al. 2013; Gijzen et al. 2016). Limited institutional support (UK Commission on Bereavement 2022) means that bereaved individuals must fend for themselves when navigating this complex terrain. Our participants had varying levels of cultural capital to draw upon to manage these processes. Some, for example, were able to draw upon cultural capital acquired through previous employment experience and their position within the field of bereavement:

I've worked in the bereavement field for many years. I worked running a children's bereavement charity. So I knew much more than most people about what to do.

(Karen, 59, bereaved wife)

Most people, however, do not have a professional background in the post‐death sector. Without this prior experience or professional insight, death administration could be difficult to manage. Participants, therefore, often utilised embodied capital acquired through their employment in non‐death related roles as leverage, drawing upon skills that they considered transferable:

I mean I do admin for a living so, in some ways, I, kind of, just…that was one of the things that I got stuck into, contacting people and making lists of things to do.

(Lauren, 47, bereaved daughter)

Capital can be accumulated, traded, and used as leverage within a field or social space (Bourdieu 1977). Participants in our study often utilised general skills acquired through the field of work‐such as organisation, communication and multi‐tasking (Sgobbi and Suleman 2015)‐ to navigate death administration. Clara's quote suggests however, that not everyone is able to draw on capital accumulated in this way:

I think the other thing as well is, she (step‐sister) probably isn’t as experienced in doing things as I am from my professional background (in academia). So, I think that, you know, she found that hard as well.

(Clara, 54, bereaved daughter)

Death administration involves a significant volume of tasks, many of which are mandatory (such as registering a death) and carry the risk of financial penalty for delay or non‐completion.^3^ It is perhaps unsurprising therefore that a deficit in cultural capital can have negative outcomes for bereaved individuals. In Bourdieu's original empirical work in education (Bourdieu and Passeron 1990), a lack of capital was directly linked to lower attainment in school. In our research, it was gaps in knowledge that lead to errors or omissions when completing administrative tasks relating to someone's death. This often resulted in bereaved individuals missing out on crucial support as illustrated by Ian, following his wife's death:

Somebody who’d been in the same position said, oh, did you ever get that bereavement grant? What grant is that? Oh, I got £1,500 or something. I said, no. Who was this from?

(Ian, 73, bereaved husband)

Ian was unaware of his entitlement to a non‐means‐tested Bereavement Support Payment available following the death of a partner.^4^ He discovered this scheme when it was too late to apply and was told: ‘no, sorry, time out… you can't have anything’. Ian's lack of specific cultural capital in this instance meant that he missed out on receiving significant financial support. Yet had Ian been able to mobilise his social capital more effectively in this situation, his lack of cultural capital would have been moot, thus demonstrating how these two forms of capital intersect with one another in the context of death administration.

Having never experienced the death of a spouse before, Ian reflects: ‘I suppose the point I'm making is it's quite easy not to know something’. Despite increased calls to improve people's death literacy (Wilson et al. 2022), through events such as death cafes (Miles and Corr 2017), there remains a pervasive reluctance to discuss issues relating to death in the Global North (Breen et al. 2022). As such, knowledge and understanding of death administration is not as easily acquired through social interaction as it is for other, more socially acceptable topics. In this sense, the taboo of death can be seen as interrupting the transference of cultural capital.

This section has illustrated how death administration processes assume members of the public have a tacit understanding about how to navigate them. But as Margaret (43, bereaved daughter) asks: ‘if you don't deal with paperwork and administration normally, how are you supposed to deal with these things?’ Through examining bereaved individuals' challenges, we have also begun to explore the links between cultural capital and death administration. Although some participants could draw on cultural capital accumulated through their position in fields such as the workplace, for many participants a lack of cultural capital was a barrier to the successful completion of death administration. In the following section we move on to explore how people drew upon social networks to acquire or access the cultural capital needed to complete death administration, emphasising the importance of adopting a relational framework to explore such areas.

### 5.2 Relationality and the Transmission of Capital Across Life and Death

Recent research has consistently shown the centrality of social relationships across death, dying and bereavement experiences (see Almack 2022; Borgstrom et al. 2019; Ribbens et al. 2023; Towers 2022, 2024). This relational understanding is largely absent, however, from literature on death administration. We seek in this section to examine the ways that participants in our study sought to mobilise their social relationships, in this instance family networks and kinship ties, to address their deficits in cultural capital (see Umamaheswar 2024 also). In doing so, they were able to make use of their social capital, combining knowledge and experience to complete death administration.

In the aftermath of loss, participants often talked about dividing tasks up among family members: ‘we all did different bits of, you know, what needed to be done’ (Howard, 59, bereaved son). This reflects Valentine's (2008) suggestion that, in response to death, family members each assume a different role and status with varying obligations and expectations. It illuminates how practical problems can be overcome when individuals are supported by multiple social ties (Lizardo 2013). In the following quote, Mark highlights the benefits and possibilities available to those able to draw upon their social capital. Mark draws on his ex‐wife's position in the legal field to complete the financial aspects of death administration:

All the other financial matters were handled by my ex‐wife who was the executor of the will… she works as a Clerk of a Court, so she’s quite good with legal stuff.

(Mark, 65, bereaved son)

Although death administration is often considered to be the responsibility of immediate family members, Mark demonstrates how a wider kinship ties can also be employed should they embody the required capital. Mark's family network entitled him to ‘the support of the collectively owned capital’ (Benzer and Reed 2019, 23), meaning that he was able to draw upon his social capital‐ and his ex‐wife's position in the legal field‐to complete certain elements of death administration.

In outlining his theory of social capital, Bourdieu (1997, 52) suggests that ‘the existence of a network of connections is not a natural or even a social given’. This can be linked with the idea of familial obligation proposed by Finch and Mason (1993). Although not referring to bereavement specifically, Finch and Mason (1993) suggest that kin relationships respond at times of crises (such as death) by offering practical and emotional support. They emphasise, however, that this assistance is fluid and operates via a pattern of guidelines rather than rules. Moreover, they claim that ‘a sense of responsibility for helping someone else *develops* over time, through interaction between the individuals… thus are *created*, rather than flowing automatically from specific relationships’ (Finch and Mason 1993, 167). In this instance, though Mark is no longer married to his ex‐wife, he remains on good terms with her and can continue to draw upon her skills and knowledge to assist him with the challenges he is facing.

Another participant, Teresa, spoke about how her father's awareness of his impending death made him start the death administration process before his physical death. This was inspired in part by his awareness of Teresa's potentially limited social capital, knowing that she had no other parents or siblings to call upon for help following his death:

Dad realised he was going to have to come and live with us and that was it, so he wanted to make it easier for me as his only child, I’ve got no siblings, and so he did his own house clearance with me. So the death admin actually started while he was still alive, because he instigated that process… I remember being in the hospice with him and he was dictating an email to the solicitor and I was typing and doing all this sort of stuff.

(Teresa, 46, bereaved daughter)

Compensating for her diminished social capital, Teresa's dad used his own cultural capital as leverage to secure the future of his only child, with the benefits of this persisting even after his death. Bourdieu (1997, 49) suggests that embodied capital ‘cannot be accumulated beyond the appropriating capacities of an individual agent’, meaning it decays with the physical and mental capacity of its owner, and yet here Teresa demonstrates how embodied cultural capital can transgress the life and death boundary. Such blurring of boundaries was visible across multiple interviews as participants were able to benefit from the knowledge and insight of their deceased relative, both prior to and following their death. Veronica's experience of completing death administration, for example, was straightforward thanks to her mum mobilising her own cultural capital prior to her death:

We knew what to do. My mum was a very practical person, so she’d constantly told us about where everything was, all the documents were. Everything was packaged up and ready to go. She’d paid for her own funeral so there was very little to do really other than going through her personal effects.

(Veronica, 59, bereaved daughter)

This section has illustrated how individuals draw on social networks to effectively navigate death administration in certain fields, thus highlighting the importance of thinking relationally in this context. The examples outlined also demonstrate how cultural and social capital intersect with one another in the context of death. More specifically, they highlight the ‘functioning of capital, the conversions from one type to another’ (Bourdieu 1986, 251), by demonstrating the circuits between the embodied and objective forms. In preparing their own paperwork, the dying relatives were able to convert their embodied capital into objective capital (often through a third‐party gatekeeper such as a solicitor or funeral provider) which those bereaved were then able to mobilise as social capital when carrying out death administration. Within this circuit, the same capital (albeit in varying forms) is transferred across the boundaries of life and death. In the final data section, we consider participants' wider relational webs by exploring how they drew upon the (paid and unpaid) institutional capital of people beyond their close familial ties.

### 5.3 The Role of Institutional Capital

Social and cultural capital, as we have sought to illuminate so far, can play a crucial role in enabling bereaved individuals to navigate death administration. There were occasions in our study, however, where participants were unable to leverage either of these forms of capital to navigate their way through the necessary tasks. This meant, as will be shown in this section, that sometimes participants needed to rely on institutional capital (i.e., third‐party and public services) and on social relationships beyond the family to enhance their understanding. As Smart (2007) highlights, it is imperative to recognise the diversity of relationships within which people are embedded, acknowledging that non‐blood kin can occupy the same position and be of equal importance as those traditionally perceived as kin (May 2011).

When participants lacked the specific cultural and social capital required to navigate death administration, they often turned to charitable third sector organisations or government agencies for assistance, as illustrated by Margaret:

I’ve spent quite a long time on the, oh what’s it called, Citizens' Advice Bureau^5^ and the government website, actually, I spent a lot of time looking through that… I was trying to find somebody that could help me, to be like, what am I supposed to do? Am I doing the right thing? Should I do this, should I do that? So that was really helpful.

(Margaret, 43, bereaved daughter)

Despite looking in the right places for information and support, Margaret explains that what she really wanted was access to someone with institutional capital, whom she could ask questions and seek reassurance from. Finding such sources of support featured in many participant interviews, and there was consistent reference to members of staff who had acted as professional ‘gatekeepers’; those who ‘control access to resources, services and knowledge’ (Collyer et al. 2017). Though locating and engaging these sources of support often still required the cultural capital to know where to look, as well as enough economic capital to access such services, with even ‘free’ websites or telephone services requiring use of the appropriate technology for access.

While certain forms of institutional capital could be acquired through public services, others (specifically legal services), were contingent on the volume of economic capital that an individual held. One financial issue regularly raised by participants was the desire to hire solicitors to help them navigate certain aspects of death administration, such as managing probate. Usually this was because the relevant paperwork was considered too complicated and participants feared that failure to complete the forms correctly would have serious repercussions: ‘I wanted a solicitor, because I was like, it's too complicated, if I mess it up, it's serious’ (Margaret, 43, bereaved daughter). As this gatekeeping operates on a monetary basis, it requires a certain level of economic capital for its acquisition, thereby reinforcing Bourdieu’s (1986, 251) argument that ‘economic capital is at the root of all the other types of capital’. For some of our participants, this meant combining resources to generate sufficient income. Yet for these people, the financial outlay was deemed necessary to acquire and utilise a solicitor's institutional capital:

The legal side of it becomes such a lot. We thought we’d try and do it on our own and pool as much information as we could together to save us the money of the solicitors but the forms are baffling so we couldn’t.

(Lauren, 47, bereaved daughter)

In the case of legal support, it was clear that participants were often required to seek out the appropriate gatekeepers. However, there were other occasions where institutional capital was voluntarily offered to participants to assist with their administrative tasks.

We have flagged elsewhere that experiences of death administration vary depending on the location and context of the person's death (Towers et al. 2023), as dying at home, for example, requires a different response and set of immediate death administration tasks than dying in a hospital. Miriam's dad died in a hospice. She was able, therefore, to draw on the institutional capital of hospice staff, utilising their position in the field of end‐of‐life care to navigate death administration: ‘They've done this a thousand times. They knew exactly which steps to take, so that was really helpful’ (Miriam, 23, bereaved daughter). The hospice workers' familiarity with death and its related administration processes meant that participants were able to benefit from the staff's institutional capital in guiding their next steps. Such assistance proved essential, as Coe (2020, 97) reflects: ‘without the support of institutional actors like funeral directors and healthcare professionals, kin do not have a cultural script of what to do’ when a death occurs in a non‐institutional setting. For participants whose relative died at home, there was often confusion around initial steps, such as whether to ring the police to report the death. Some of these participants mentioned the role of individual professionals who had offered them significant guidance and insight:

I was very grateful to have her there (when he died) it was good really because once I'd had my time with him, then she (Macmillan nurse^6^) was able to say, right, well we need to move him now because he was sat up when he died. And she explained the process and everything.

(Fiona, 58, bereaved daughter)

Although Fiona had been in regular contact with the Macmillan nurse before her father's death, it was only at the time of his death that she had needed to draw upon the nurse's capital to inform her own actions. In this sense, her father's death represented a point of ‘activation’, reflecting the understanding that ‘social ties are transformed from a potential store of resources into actual social capital when persons can accrue benefits from those ties’ (Lizardo 2013, 321).

Much like the healthcare professionals in Collyer et al. ’s (2017) study, these professionals were able to apply their institutional capital to shape the experiences of those in their care:

She (nurse) said to me… if she passes tonight then I want you to do two things for me… don’t phone the doctor, phone me, because your wife doesn’t want anybody turning on her chest and trying to recover her… Secondly, if she dies tonight, and when she does, immediately call the undertaker and arrange a time for her body to leave… I was probably in a bit of a state already, but I did listen to those words of counsel.

(Ian, 73, bereaved husband)

Nurses therefore appear to possess clear system knowledge (Willis et al. 2016), which in Bourdieu's terms, means they are familiar with local systems and services, but crucially, they also understand how they work. These gatekeepers have an awareness of the field and demonstrate an understanding of the ‘rules of the game’ (Bourdieu 1984, 471). As Willis et al. (2016) found within the healthcare sector, possession of this system knowledge is integral to navigating death administration processes. Both Fiona and Ian were keen to express their gratitude to these professionals for sharing their institutional capital and helping with their first administrative steps, thereby demonstrating the value of these time and context‐bound social relations that feature within an individual's relational webs. Interview quotes presented in this section therefore reinforce the importance of thinking relationally in the context of death administration, emphasising the importance of recognising social connections beyond the family. It also illuminates how economic capital can inform people's access to institutional resources, thus reinforcing Bourdieu's argument on the underpinning role that economic capital plays (Bourdieu 1986).

## 6 Conclusion

Death administration requires specific knowledge and skill for its completion. As illustrated throughout this paper, bereaved individuals have varying types and qualities of capital that they can draw upon to manage these processes. Such capital, as acknowledged, is informed by an individual's habitus and is accumulated, traded and used as leverage within specific social spaces. This article has illustrated how social and cultural capital intersect to influence the ability of bereaved people to complete death administration, highlighting the centrality of social relations in this process. In doing so, the paper has extended the focus of existing socio‐theoretical discussions about the role and use of capital and contributed new knowledge to sociological understandings of the social relations of death, dying and bereavement.

Institutional lack of clarity, as highlighted, often displaces responsibility from organisations onto bereaved individuals, potentially affecting their mental health and wellbeing (Reed and Balazs 2024). These harmful consequences can be exacerbated by disparity in bereaved individuals' access to the social, emotional, and material resources required to manage death administration, as well as the support services designed to help navigate this process. There is a distinct inequity of experience and outcome for people depending on their cultural capital (gathered through professional and personal experience), their social capital (the social networks available to them) and the professional gatekeepers they encounter (as well as the economic capital available to mobilise these professionals). By illuminating the extensive and intersecting impact of capital in death administration processes in this way, the paper offers novel insight into how capital operates in certain hidden areas of society.

Bourdieu's theory of cultural capital (1986) has been used extensively to examine social phenomena in the context of life (Lareau and Weininger 2003; Sullivan 2002). Less is known about how it operates in the context of death. Death marks the end of biological life. As we have highlighted, however, it does not always indicate the death of embodied capital. Instead, death can prompt a transference of capital, that can be followed through a series of circuits that are, at times, operated by gatekeepers. And yet, death remains an awkward topic of conversation and so these conversions remain hidden. This paper brings to light these shifts in capital, using the context of death administration to explore how individuals draw upon social ties to share and transfer capital, sometimes across the life and death boundary. In doing so the paper offers an original contribution more specifically to sociological debates on embodied cultural capital.

Research on one aspect of death administration‐funeral planning‐illustrates the relational nature of this activity (Woodthorpe and Rumble 2016). Wider research on death administration processes has, however, neglected to account for the way that bereaved individuals are embedded in a range of social networks. Death administration, as we have shown in this article, is a relational activity. Our qualitative data highlights the importance of locating bereaved individuals within their wider webs of relationships (of varying sizes) when considering how best to support them. Acknowledging this is an important step forward in discussions of how to address the inequity of capital that people must draw upon when completing death administration.

One of the authors has argued elsewhere (Reed and Balazs 2024) that inconsistent and contradictory bureaucratic processes have a strong presence in death administration, often constituting bureaucratic violence‐a mundane but pernicious form of violence based on exclusionary organisational processes which can serve to reinforce rather than transcend social division. Although our research participants were from exclusively middle‐class and professional backgrounds, the fact that a group of people with generally higher levels of social, cultural and economic capital found death administration so difficult is problematic; it suggests that these issues will be even more prevalent among those who are more financially and socially vulnerable. This highlights a pressing need for further research with a wider range of demographic groups, with the aim of developing a more accessible and transparent set of death administration procedures. This is essential if we are to address the inequalities inherent within post‐death processes.

## Conflicts of Interest

The authors declare no conflicts of interest.

## Data Availability

The data that support the findings of this study are available from the corresponding author upon reasonable request.

## Endnotes

^1^
For example, the gov.uk website states that ‘a relative should register the death’ with strict requirements for any non‐relatives seeking to register a death instead.

^2^
The National Bereavement Service is a UK based, not‐for‐profit organisation that provides practical and emotional assistance after a death.

^3^
A death in England must be registered within 5 days, including weekends and bank holidays. Failure to do so may result in a fine or delays in obtaining further documentation.

^4^
This is subject to certain eligibility requirements concerning, for example, the age of the person when their partner died, the status of their relationship, and the deceased's total National Insurance contributions.

^5^
The Citizens Advice Bureau is a network of charities in the UK that provides free, independent and confidential advice to people.

^6^
Macmillan nurses specialise in providing care and support for individuals living with, and dying from, cancer.
